# Communication about early palliative care: A qualitative study of oncology providers’ perspectives of navigating the artful introduction to the palliative care team

**DOI:** 10.3389/fonc.2022.1003357

**Published:** 2022-12-09

**Authors:** Anna Collins, Lorna Gurren, Sue-Anne McLachlan, Olivia Wawryk, Jennifer Philip

**Affiliations:** ^1^ Department of Medicine, St. Vincent’s Hospital, University of Melbourne, Melbourne, VIC, Australia; ^2^ Department of Medical Oncology, St. Vincent’s Hospital, University of Melbourne, Melbourne, VIC, Australia; ^3^ Parkville Integrated Palliative Care Service, Peter MacCallum Cancer Centre and The Royal Melbourne Hospital, Melbourne, VIC, Australia

**Keywords:** early palliative care, oncology, referral, communication, qualitative study

## Abstract

**Background:**

Despite robust evidence for the integration of early palliative care for patients with advanced cancer, many patients still access this approach to care late. Communication about the introduction of Early Palliative Care is an important skill of healthcare providers working in this setting. In the context of limited community understanding about palliative care, patients and their families may express fear or negative reactions to its early introduction. Health professionals may lack the confidence or skill to describe the role and benefits of early palliative care.

**Aim:**

This study sought to explore clinicians’ perspectives on communication about referral to early palliative care, specifically identifying facilitators in undertaking this communication task.

**Methods:**

An exploratory qualitative study set within a tertiary oncology service in Victoria, Australia. Semi-structured interviews were conducted with purposively sampled oncology clinicians exploring their perspectives on communication about referral to early palliative care. A reflexive thematic analysis was undertaken by two researchers, including both latent and semantic coding relevant to the research question. Reporting of the research was guided by the Consolidated Criteria for Reporting Qualitative Research (COREQ) checklist.

**Results:**

Twelve oncology clinicians (58% female, with 67% > 15 years clinical experience) from medical oncology, surgical oncology, and haematology participated. The artful navigation of communication about early palliative care was characterised by the need for a ‘spiel’ involving the adoption of a series of strategies or ‘tactics’ when introducing this service. These themes included: *1) Using carefully selected and rehearsed language; 2) Framing in terms of symptom control; 3) Framing as additive to patient care; 4) Selling the service benefits of early palliative care; 5) Framing acceptance of referral as an altruistic act; and 6) Adopting a phased approach to delivering information about palliative care.*

**Implications:**

This study highlights the wide ranging and innovative communication strategies and skills required by health professionals to facilitate referral to early palliative care for cancer patients and their families. Future focus on upskilling clinicians around communication of this topic will be important to ensure successful implementation of models of early palliative care in routine cancer care.

## Introduction

Palliative care, concerned with the relief of physical, psychological, social, and spiritual suffering (1), is associated with improved clinical outcomes for patients and their families. Several meta-analyses demonstrate benefits for patients including greater health-related quality of life, reduced symptom burden, improved mood, and even prolonged survival (2–4). As such, there is growing impetus to integrate palliative care earlier in the cancer care pathway (5), reflected in the ASCO guideline that patients with advanced cancer receive dedicated palliative care services concurrent with anticancer treatment (6). Yet, late referral of cancer patients to palliative care specialists continues to be identified across international settings (7–9).

The realisation of early integration of palliative care is hampered, in part, by the unique communication barriers in this context (10, 11). Communication between patients and clinicians is a relational process underpinning all oncology and palliative care (11). Communication is broadly considered a core determinant of quality end-of-life care (12), and has ensuing implications for the health of the caregiver in bereavement (13). Despite population-level preferences of >70% who want to be informed about options regarding palliative care in the event of serious illness such as cancer (14), patients and their caregivers report inadequate communication about palliative care, including a tendency to use euphemistic or technical language that is difficult to understand (15, 16). Underlying such challenges in care are the clinician-perceived communication barriers related to the introduction of palliative care (17).

Among these clinician-reported communication barriers are a fear of diminishing patients’ morale (18), prognostic uncertainty (19, 20), perceived lack of adequate training for such discussions (21, 22), language and cultural factors (17), and difficulty judging the appropriate time for these discussions (23). These factors are compounded by variable levels of community understanding about palliative care (24), with perceptions of relevance only for those imminently dying (25), meaning patients may also be fearful or avoidant of discussing early palliative care (15). Thus the communication landscape in the setting of early palliative care is fraught, with communication paradoxically representing both a core component of and barrier to early integration with oncology (26).

While the introduction of ‘Early Palliative Care’ is an important skill of clinicians working in the advanced cancer setting, there has been limited empirical focus on communication facilitators or strategies to navigate this specific task (27). This task is one largely reliant on professionals who are not routinely trained in this specific aspect of care and whose core focus is a different specialty (22). In short, the referral communication task occurs ‘outside’ the field of palliative care where many have the training and skill sets to undertake such complex conversations.

While communication skills training for cancer care clinicians appears effective in improving support for patients in consultations such as when “difficult news” is delivered, uptake of such training is not yet widespread (26, 28–31). As such, the informal, self-adopted strategies of clinicians therefore remain particularly relevant. This study sought to explore oncology healthcare professionals’ perspectives on communication about referral to early palliative care, specifically identifying facilitators when undertaking this communication task.

## Methods

### Study design

This study employed an exploratory, qualitative, cross-sectional design using semi-structured interviews to elucidate oncology clinicians’ perspectives on communication of a palliative care referral. The epistemological position adopted by the researchers reflects social constructionism in which positivist notions of mapping reality in a decontextualized sense were rejected in favour of a view of knowledge that is circumscribed, in part, by social context (32). Methodological rigour was conceptualized in line with Lincoln and Guba favouring trustworthiness (transferability, dependability, credibility, confirmability) (33) over quantitative notions of reliability and validity (34). Activities to enhance trustworthiness included the following: AC and LG (both researchers in palliative care, and experienced in qualitative analysis) engaged in an ongoing process of reflexivity through co-analytic sessions during which varying interpretations of the data were questioned and challenged; an audit trail of the data analytic process was kept; and diverse participant perspectives were triangulated *via* a purposive sampling framework. The reporting of this research is consistent with the Consolidated Criteria for Reporting Qualitative Research (COREQ) checklist (35).

### Ethics

Ethics approval was provided by the Institutional Human Research Ethics Committee in St. Vincent’s Hospital Melbourne (LRR 070/15). Informed consent was obtained from each participant prior to study participation.

### Study participants and setting

Participants were recruited through the oncology service of a tertiary hospital in the metropolitan centre of Melbourne, Victoria, Australia. A purposive sampling strategy was utilised to identify a group of clinicians currently providing care to patients with advanced cancer who routinely required referral to palliative care. Purposive sampling ensured that both male and female perspectives were included in the sample, in addition to representation across oncological specialties, and a range of clinical experience. No participants who were invited declined participation.

### Data collection

Data from participants were collected by one researcher (AC) using semi-structured interviews (n = 4) and focus groups (n = 8) of 40-65 minutes duration. These were conducted face-to-face in the hospital setting during 2018. Interviews were included to allow broader participation where purposively sampled participants were unavailable at the focus group times. Demographic information on participants was collected *via* a brief survey including gender, profession, and number of years’ clinical experience. The interview guide (**Table 1**) used for all data collection included an initial open exploration of clinicians’ experiences with referring patients to palliative care. Subsequent questions related to views of the timing of palliative care referral, communication about palliative care, and barriers and enablers relating palliative care referral. Supportive affirmations and direct probes were used to encourage dialogue and prompt further discussion around these topics of interest. In the present research, data on communication about palliative care were analysed and are presented herein, with other procedural and systemic barriers to palliative care referral to be published elsewhere.

**Table 1:** Interview guide.

Topic	Question	Prompts, as needed
*PREAMABLE: Thank-you for giving up your time today to attend this focus group/interview. You may recall the purpose of the session is to discuss issues pertinent to the introduction of early palliative care for patients with advanced cancer and their families.*		
**Topic 1: Discussion of past cases when the clinician has referred a person for early palliative care**	*To begin, can you reflect back upon a previous patient that you have referred to early palliative care. Please discuss this case and share some of the circumstances leading up to this referral, the discussion that took place, and how this was received.*	What prompted the referral/conversation? How was the referral/conversation received? How did the patient and their family respond? Did you feel it was appropriate timing? If so/why? Why not earlier? Why not later?
**Topic 2:** **Views of the timing of palliative care referral early in the illness**	*Now I would like to discuss how you see early palliative care fitting into the overall management and support offered for patients with advanced cancer. How would you define the timing of ‘early’ palliative care?*	What are the key needs and concerns at the time of introduction to early palliative care? - For patients? - For families? - For you as a health care provider? At what time in the person’s illness might you consider referring to early palliative care? What prompts you to think you should get palliative care involved early on?
**Topic 3:** **Communication about early palliative care**	*I wonder now, if we can discuss, how you might go about talking about early palliative care with patients and their families once you’ve you identified they may benefit from it.* *Given some of the complexities of talking about early palliative care, how might you explain this to patients and their families?*	What might you say? What works well? What doesn’t work so well? What strategies do you use to introduce early palliative care? What is difficult about this discussion?
**Topic 4:** **Barriers and enablers relating early palliative care referral.**	Lastly I would like to discuss your views of the barriers and enablers associated with referral to early palliative care. When you see a role for early palliative care, what do you see to be the key inhibiting and enabling factors in raising this?	Patient understanding of palliative care? ▪ If so/what do they understand Patient hopes Communication Time Relationships Others?

### Data analysis

Audio recordings were transcribed verbatim, de-identified, before being subjected to analysis. The researchers adopted an inductive, reflexive approach to thematic analysis, using a combination of latent and semantic codes (36, 37). This reflexive process first involved initial data immersion and familiarization through repeated reading of the transcript and initial coding to identify latent content within the data. Subsequently, these codes were further abstracted to identify conceptual similarities and differences between codes with related codes then clustered together. Codes that were not coherent in the context of the meaningfulness across the dataset or irrelevant to the research aims were removed. Themes were constructed with the remaining related codes, and themes were then defined and refined to ensure appropriate wording. The write-up process then ensued with the use of illustrative quotations accompanying the themes presented. Trustworthiness in the analytic process was ensured through ongoing meetings between L.G and A.C, to discuss and justify the phrasing and content of codes, and the conceptual relations and organization between codes, themes, and subthemes. This process reflected peer debriefing, which can enhance the credibility of the analysis (33).

## Results

### Sample description

Twelve oncology clinicians (58% female) from medical oncology (n = 8), surgical oncology (n = 3) and haematology (n = 1) participated (**Table 2**). Eight (67%) of the participants reported over 15 years in practice, with 4 (33%) who reported less than 15 years’ experience.

**Table 2:** Characteristics of participating health care professionals.

	N=12	%
**Gender**		
Male	5	42
Female	7	58
**Discipline**		
Medical oncology	8	67
Haematology	1	8
Surgical oncology	3	25
**Years of experience in discipline**		
< 5	3	25
5-15	1	8
15+	8	67
**Focus group or interview**		
Focus group	8	67
Interview	4	33

### Findings - artful navigation of palliative care conversations: *“You have to tiptoe around it”*

The artful navigation of communication about early palliative care was characterised by the need for a ‘spiel’ involving the adoption of a series of strategies or ‘tactics’ when introducing this service. Healthcare Professionals (HCPs) described the various techniques they carefully executed to skillfully introduce the concept of ‘early palliative care’ to patients with advanced cancer. These strategies reflected clinicians’ attention to the timing of the introduction, the quantity of information presented, and the specific content or framing of the message itself.

Six constructed themes represented healthcare professionals’ perspectives on these key strategies for navigating the communication landscape in palliative care referral, namely: *Using Carefully Selected and Rehearsed Language; Framing Palliative Care in Terms of Symptom Control; Framing Palliative Care as Additive to patient care; Selling Service Benefits of Adding Palliative Care to Standard Oncology; Framing Palliative Care Referral Acceptance as an Altruistic Act;* and *Adopting a phased approach to delivering information about palliative care.* Each theme is described in turn, with illustrative examples from the clinicians’ data.

#### 1. Using carefully selected and rehearsed language: *“Mention the ‘palliative care’ word and you can see the face drop”*

HCPs in this study described their carefully selected and rehearsed language in consultations where they sought to introduce early palliative care to patients with advanced cancer. In particular, this related to clinicians’ perceived need to avoid the term ‘palliative care’ when discussing referral with patients:

“Mention the ‘palliative care’ word and you can see the face drop”.

The need to avoid the use of this term was described in relation to negative, patient-held connotations of this name, and specifically end-of-life connotations:

“As soon as you mention ‘palliative care’, people think you’re talking about end of life”.

A variety of strategies were proposed by HCPs for managing or circumventing this anticipated issue in the referral process. Commonly, clinicians described the strategy of first dispelling patient-held negative connotations before using the term. One HCP noted that they preface the use of this term with an instruction for the patient to disregard any end-of-life preconceptions they have about this service:

“I’ve been prefacing it by saying ‘I want you to ignore the terminal connotations of this referral’”

Another HCP described the strategy of delaying the use of the ‘palliative care’ term until late during the referral discussion, owing to the fact that patients’ families do not engage with the remainder of what the clinician has to say, once they hear the word:

“Often I don’t mention the word ‘palliative’ until … late … because I think that once a patient’s family hears the word ‘palliative’ they have an immediate impression. And [I find] they almost don’t hear the conversation, if you’re introducing it.”

In the absence of a different name for palliative care, some clinicians described favouring an approach to ‘get them in the door’ by introducing the concept of early palliative care using different terms. Some clinicians adopted the strategy of simply avoiding the term ‘palliative care’ completely:

“I don’t even mention palliative care.”

Related to this, several HCPs in this study raised the suggestion of changing the name of ‘palliative care’ in general:

“They should just change the name of ‘palliative care’ anyway.”

#### 2. Framing palliative care in terms of symptom control: *“It’s for us to manage your symptoms a little bit better”*

HCPs in the study described framing Palliative Care in terms of symptom control when introducing early palliative care with patients. The approach of directly aligning palliative care with symptom control involved characterizing this service as the best approach to manage the symptoms that the person is presently experiencing:

“I say, ‘Look, you’ve got symptoms. The best people to manage symptoms are the palliative care service.’ And I actually … frame it in the symptom management kind of way.”

Similarly, two participants noted that they specifically frame the palliative care service in terms of being the “pain team” or as specialists in pain control:

“I think I found it easy to introduce palliative care as the pain specialists.”

HCPs noted that they opt for the term ‘symptom control’ specifically due to the stigma associated with the term palliative care:

“I think you have to frame it [palliative care] as symptom control. I think it’s like a stigma.

The approach of framing palliative care as symptom control was therefore perceived by HCPs as an effective strategy, describing that it was helpful to avoid negative patient-held reactions:

“Because I frame it a bit differently, maybe I don’t see so many people reacting [negatively] so much now.”

However, one HCP in the sample noted that, when patients are asymptomatic, this makes it difficult to discuss PC:

“I guess I would have difficulty broaching, or more difficulty broaching pall(iative) care when a patient has no symptoms whatsoever from their cancer.”

Indeed, one HCP expressed skepticism with respect to the effectiveness of symptom control framing, noting that irrespective of the framing used, palliative care will hold different connotations for some patients:

“It’s easy to say ‘it’s just how you sell it’ but that’s not really true. I mean, yes, it’s how we’re going to get a group of people who can help us see if we can … to remove the pain or whatever it is. But it still sends a slightly different message to a lot of people.”

#### 3. Representing palliative care as additive to patient care: *“[Palliative Care] is about adding extra things in. It’s not about taking things away”*

In introducing early palliative care, HCPs in the study described representing this concept as an additional layer of support to their care. One HCP described explaining to patients that it involves adding elements to their care, as opposed to withdrawing care:

“I sort of emphasize that [palliative care] is about adding extra things in. It’s not about taking things away”

In this context, this participant also noted a perceived obligation to reassure their patients that a referral for early palliative care does not mean discontinuation of their oncologic care:

“I feel I’m obliged to say ‘Just because you go to palliative care doesn’t mean I’m going to stop seeing you.’”

Moreover, HCPs raised their strategy of explicitly offering the patient a choice of which professionals are involved in their care, in this way framing the addition of palliative care to the team of professionals to work with the oncologist:

“I say, ‘Look, do you want me to be involved? Would you prefer me to involve somebody fresh and new who specializes in this area? Would you prefer us to work as a team?”

One HCP also noted that they frame palliative care professionals as ‘experts’ who are even ‘better’ than the referring clinician, being an added pair of eyes who can make better recommendations for care:

“I’ve found it’s good to sell it as them being experts even better than me. I’ll say ‘They are a lot better than me at this. I could prescribe something, but an extra pair of eyes, they can find key things, or suggest something better.’”

#### 4. Selling service benefits of adding palliative care to standard oncology: *“Usually what I’m talking about is services”*

HCPs noted how they inform patients of the specific service benefits of adding palliative care to their standard oncologic care. One HCP described framing palliative care in terms of necessary services which could solve existing anxieties or concerns of patients and their family members:

“Usually what I’m talking about is services, and trying to identify things that family might already be worried is going to be a problem.”

Another HCP enumerated several specific service benefits of palliative care including the role to facilitate patients staying at home or out of the hospital, or if admitted, to align with a person’s desire to go home:

“I sort of sell the role of palliative care in discharge planning. The importance of not just going home, but being comfortable at home, being able to stay home for a significant amount of time.”

The availability of advice or care 24 hours a day and seven days a week was another specific service benefit described by HCPs as a strategy when describing early palliative care to patients. Palliative care was framed as the point of contact for patients and their family if they found themselves at home with concerns and needing help, in this away avoiding the emergency department:

“Another selling point I’ve used is, let’s say you’re at home and your symptoms get worse. It’s nice to have someone to call, people who can visit, rather than coming back to ED waiting for hours.”

The ‘pace’ of palliative care was also described by one HCP as a selling point used when discussing palliative care with patients, whereby they note that the service affords more time for care:

“I sell the pace. I say you won’t be rushed on the pall care ward.”

#### 5. Framing palliative care referral acceptance as an altruistic act: *“We’re not just doing this for you. We’re actually doing it for your wife”*

Several HCPs described framing early palliative care as a service needed by family members or even by themselves as their health professional when attempting to convince patients to accept an early referral to palliative care. Two HCPs noted that family members are eager to avail themselves for support:

“I think the partner is desperate for help…”

This HCP notes that such help for a patient’s partner may be provided by palliative care:

“[palliative care] is support for the partner.”

Similarly, HCPs on occasion noted that they inform patients that, by accepting an early referral to palliative care, they would help them as their doctor, knowing that they have support in the community who can contact them when needed:

“I often say that ‘it will be helpful for me if we organize this.’ I say it makes my life much easier if I know you’ve got this support in the community and they can contact me when they need to. And it would be helpful for me.”

#### 6. Adopting a phased approach to delivering information about palliative care: *“They get so much information in the beginning, and they just haven’t got a clue”*

HCPs discussed the benefits of raising palliative care early, when it is ‘not needed’, giving an opportunity to address misperceptions. At the same time, HCPs also perceived that patients with advanced cancer are presented with copious amounts of information at diagnosis. In this context, HCPs described difficulty in introducing the discussion of palliative care early following diagnosis, owing to the perception that it may be overwhelming for the patient:

“I personally find I just explained the cancer alone, even without getting treatment, that might be too overwhelming at first consultation. No doubt they need pal care, but personally I feel like maybe it might be too early [to discuss]”

One HCP noted that this is particularly true of younger cancer patients as, in their perspective, these patients find palliative care particularly difficult to understand at this initial stage:

“We’re increasingly seeing these younger patients with metastatic cancer … I just find bringing up pal care at [the initial] stage is even harder for them to comprehend.”

Related to this, another HCP described not introducing the topic of palliative care at diagnosis, but wait for the consultations that follow:

“Maybe within a time frame so get over the period where they’re just taking cancer as a word, digesting the treatment. And just have a person say within two or three weeks to find them”.

When introducing the conversation about early referral to palliative care, HCPs described the careful balancing act of the need for providing the patient with information about the service, while at the same time recognising that too much detail may also lead the patient to close the conversation. In response, some HCPs adopted the strategy of introducing the concept of palliative care in broad terms only:

“I start to maybe outline some of the things that pall care can offer, but maybe not in a lot of nitty-gritty details”.

Responding to this tension, some HCPs also described the strategy of delivering the introduction to palliative care in a piecemeal or ‘drip feed’ approach, involving a brief mention in one consultation and then following up at a later visit:

“I find it’s good just to sort of plant the seed [of palliative care] one time, follow it up the next time. And it might actually be two or three visits before you actually get them to say ‘yes’”.

## Discussion

With the implementation of early palliative care far from being standard of care across many international settings, communication around the task of referral is one key issue that requires further attention. This study sought to explore oncology clinicians’ perspectives on communication about palliative care, highlighting some key strategies adopted to underpin the content, framing, and timing of the communication about early referral. The ‘artful’ navigation of introducing early palliative care, characterised by the need to employ various strategies executed overtime to enable referral, point to the complexity of this clinical communication task. To ensure the successful implementation of models of early palliative care in routine cancer care, further empirical studies to distill the effectiveness of these strategies, and interventions to support clinicians around communication of this topic are needed.

This study has revealed strategies adopted by oncology clinicians who must frequently broach referral to early palliative care. These strategies were largely focused around referring clinicians’ perceived need for a rehearsed ‘spiel’ to introduce this concept in a manner perceived to be ‘gentler’, ‘easier’ or more palatable for patients. This involved carefully selected words and framing of palliative care: for symptom control; as experts – mostly in pain management; to help loved ones; to help the treating clinician; to add to the care team; to access specific services or tasks; to support patient hopes to go home; to support a focus on quality of life. Interestingly, some of this framing is also consistent with the palliative care discourse which has seen messaging used by palliative care professionals focused on actions (e.g. availability, family care, wellbeing), values (e.g. individualized care – ‘you matter’) or alignment with immediate needs (e.g. support to go home), rather than identity (11, 38).

The approach used by clinicians also involved considered timing as to when to optimally raise this discussion – timing not necessarily defined based upon the best evidence but instead framed around enhancing patient acceptability through a phased, ‘drip-fed’ approach. This has resonance with a stepwise questioning strategy observed in other studies, allowing opportunities for the person to engage in difficult talk without explicitly inviting such talk or placing patients in this potentially delicate position (39).

The finding that clinicians avoid using the term ‘palliative care’ is consistent with prior literature indicating stigma associated with this term (10, 15, 16, 40) and that many referring clinicians and patients dislike and opt to avoid this term (15), frequently in favour of re-branding as ‘supportive care’ (41–45). In this study some clinicians also broadly suggested that the name of the discipline should be changed. The extant debate in the literature regarding the need to re-name ‘palliative care’ has seen a spectrum of perspectives. This includes proponents, often from referring specialities, who argue that a name change is necessary due to the stigma associated with the name.

Conversely, opponents, often from palliative care, point to the risk of a ‘euphemistic treadmill’ (46), citing the limited (or short-term) increase in palliative care referral following a name change in a given setting (47) or arguing that the limitations of the term are a cultural artefact and hence would not be readily ameliorated by a name change (48, 49). Indeed surveys of palliative care clinicians suggest that although many recognise patients perceive the term “palliative care” negatively, few believe a name change to supportive care would encourage early referral, and <21% support renaming the specialty (7, 50). Others point to the opportunities for a re-branding of message which conveys the benefits, re-focuses the message, and builds the service accordingly, while still maintaining the name, palliative care (51–53). In any case, the results of this study highlight the complexity of palliative care discourse and potential for mixed messaging underpinning ‘early palliative care’ (38, 49, 51). Further, the results support the need for greater clarity of message within the communication underpinning referral to early palliative care, explaining the intended role and service offerings to health professionals and patients (10).

While clinicians spoke of using different terms and strategies to avoid patient distress while enabling palliative care referral, it is possible that they are also seeking to avoid their own discomfort. While there is little literature in the area, the use of euphemisms, different names, and discussion of activities of palliative care such as pain relief enable the patient and the physician to avoid talking of end-of-life care and death (15). It is possible that the use of this avoidant strategy serves to not only protect patients from distress, but also to lessen their own discomfort in discussing palliative care and its implications. Exploring this possibility and undertaking to directly evaluate clinicians’ distress in performing an early referral may provide opportunities for redress. Importantly, these communication aspects, as a seemingly major barrier, will be crucial to address in any future implementation of systematic screening for palliative care referral. The finding in this study of framing palliative care referral acceptance as an altruistic act is consistent with the literature on oncology trial participation. Such research has demonstrated that altruism is a frequent motivation for clinical trial participation (54), with patients demonstrating high agreement for scale items such as ‘contributing to research that can help others in the future’ as a motivation for their participation. However, cancer patients are not monolithic in these altruistic motivations, with patients with a poorer prognosis demonstrating a lesser altruistic motivation for research participation than for those with a better prognosis (55). There is a dearth of literature exploring the role of altruism in palliative care referral acceptance among cancer patients. However, it is conceivable that the framing of referral acceptance in terms of supporting others (e.g., family, primary clinician) may capitalize on altruistic motivations among cancer cohorts and thereby facilitate referral. Further research is necessary to investigate altruism as a motivator of palliative care referral acceptance in this cohort. Potential ethical implications of this framing, for example in terms of coercion, must also be explored.

## Study strengths and limitations

When exploring the nature of communication in healthcare, observational research methods such as conversation analysis offer an alternative approach which may elucidate different issues. Additionally, triangulation of the findings with a patient sample would further add to the interpretation, which in this analysis, was limited to the clinician’s perspectives of how they communicate about early referral to palliative care. Similarly, other referring clinicians such as General Practitioners working in community settings may provide additional important perspectives around this task. Finally, the prior communication skills training of clinicians participating in this study was not recorded. Such training has been demonstrated to influence cancer care consultations and would likely enhance confidence and practices in this important task of introducing early palliative care. Nonetheless, this well-designed and analysed qualitative study, albeit small, provides new insights into the strategies used by clinicians in this communication task which can form the basis of further study, highlight the role for formal communication skills training, and support other clinicians seeking peer guidance on introducing early palliative care.

## Conclusion

This study reveals the complex task of communicating about early referral to palliative care and the communication skills required by health professionals to ‘artfully’ navigate this task. Oncology clinicians conveyed their self-adopted strategies which underpinned their ‘spiel’ to ease the introduction of early palliative care for patients and their families, and perhaps for the clinicians themselves. This was characterised by careful rehearsed language, framing and attention to the timing of the introduction- none of which was necessarily straightforward or overt. While there are apparent opportunities and also limitations of such an approach, the attention given to this clinical task equivalent to a “breaking bad news” conversation gives weight to its importance. The future successful implementation of models of early palliative care integration in oncology will require support for communication skills training specific to introducing early referral to palliative care.

## Data availability statement

The raw data supporting the conclusions of this article will be made available by the authors, without undue reservation.

## Ethics statement

The studies involving human participants were reviewed and approved by St Vincent’s Hospital Melbourne Human Research Ethics Committee. The patients/participants provided their written informed consent to participate in this study.

## Author contributions

AC was the lead investigator, obtained the study funding, was responsible for the study conduct, had access to the data, and co-authored the first draft. AC, LG, and OW were involved in the data analysis. All authors contributed to the study protocol, interpretation and, contributed to the manuscript. All authors contributed to the article and approved the submitted version.
